# Diffusion-weighted MRI-derived ADC values reflect collagen I content in PDX models of uterine cervical cancer

**DOI:** 10.18632/oncotarget.22388

**Published:** 2017-11-11

**Authors:** Anette Hauge, Catherine S. Wegner, Jon-Vidar Gaustad, Trude G. Simonsen, Lise Mari K. Andersen, Einar K. Rofstad

**Affiliations:** ^1^ Group of Radiation Biology and Tumor Physiology, Department of Radiation Biology, Institute for Cancer Research, Oslo University Hospital, Oslo, Norway

**Keywords:** DW-MRI, ADC, collagen, cervix carcinoma, PDX models

## Abstract

Apparent diffusion coefficient (ADC) values derived from diffusion-weighted magnetic resonance imaging (DW-MRI) are known to reflect the cellular environment of biological tissues. However, emerging evidence accentuates the influence of stromal elements on ADC values. The current study sought to elucidate whether a correlation exists between ADC and the fraction of collagen I-positive tissue across different tumor models of uterine cervical cancer. Early and late generation tumors of four patient-derived xenograft (PDX) models of squamous cell carcinoma (BK-12, ED-15, HL-16, and LA-19) were included. DW-MRI was performed with diffusion encoding constants (*b*) of 200, 400, 700, and 1000 s/mm^2^ and diffusion gradient sensitization in three orthogonal directions. The fraction of collagen I-positive connective tissue was determined by immunohistochemistry. Mono-exponential decay curves, from which the ADC value of tumor voxels was calculated, yielded good fits to the diffusion data. A significant inverse correlation was detected between median tumor ADC and collagen I fraction across the four PDX models, indicating that collagen fibers in the extracellular space have the ability to inhibit the movement of water molecules in these xenografts. The results encourage further exploration of DW-MRI as a non-invasive imaging method for characterizing the stromal microenvironment of tumors.

## INTRODUCTION

Diffusion-weighted magnetic resonance imaging (DW-MRI) is a non-invasive imaging method of increasing interest in the field of cancer. The technique exploits the thermally driven motion of water molecules in tissue, with image contrast arising from the varying microscopic mobility of water in different microenvironments [1, 2]. Tumors are known to exhibit an abnormal microenvironment, often characterized by high cellularity, a dense interstitial structure, and accumulated solid stress [3]. Such abnormal features have led scientists and clinicians to hypothesize that parameters derived from DW-MRI may have the potential to serve as cancer biomarkers. Moreover, as this imaging technique does not require any exogenous contrast agent, does not use ionizing radiation, and can be performed quite rapidly, the hypothesis is worthy of investigation.

The most commonly derived parameter from DW-MRI is the apparent diffusion coefficient (ADC). This quantitative parameter describes the observable – or “apparent” – diffusion of water molecules within tissues, which is typically several-fold less than in pure water [2]. *In vivo*, the effective displacement of water molecules is highly influenced by the cellular environment, including the density and type of cells, their organization, and the intactness of their hydrophobic membranes [1]. Malignant lesions with high cellularity are therefore expected to have lower ADC values than their benign counterparts or normal tissues. This has been confirmed by a range of studies [4–6], although the presence of cystic or necrotic regions (presenting with high ADC values due to unrestricted diffusion) may give rise to false-negative results [1]. Significant correlations between ADC values and cell density have been detected for several tumor types [5, 7–10]. However, for some malignancies, correlations with cellularity have been weak or absent [11–13], indicating that *in vivo* water diffusivity is not solely explained by the cellular component of biological tissues.

Tumor tissue is composed of malignant parenchymal cells embedded in a supportive connective tissue basis; the tumor stroma. The stroma comprises blood vessels, immune cells, and activated connective tissue cells, *e.g.* fibroblasts, producing extracellular matrix (ECM) constituents, such as polysaccharides and various fiber proteins [14]. The main structural protein in mammalian connective tissues is collagen, of which type I collagen is the most abundant [15]. Tumor cells tend to interfere with the normal biosynthesis of the ECM, resulting in aberrant production and distribution of fibrous components of the tumor stroma [16, 17]. This has implications for cancer therapy, since collagen and other ECM proteins may serve as steric barriers to the interstitial diffusion of therapeutic agents [3, 18]. Moreover, a variety of signaling pathways has been reported to be influenced by collagen changes in the tumor microenvironment. These affect angiogenesis, cell migration, tumor invasiveness, and – consequently – the clinical outcome of cancer patients [19].

With increased appreciation of the role of the stromal microenvironment in tumor progression comes the desire for robust biomarkers, yielding information on the amount and distribution of connective tissue in tumors. Our group has previously discovered that ADC values are inversely correlated with the fraction of collagen in individual tumors of the CK-160 model – a cell line-derived xenograft model of uterine cervical cancer [20]. We therefore hypothesized that DW-MRI can provide information on the tumor stroma across several biologically different cervical cancer models. The present study aimed to investigate whether ADC values are associated with collagen I fraction in four patient-derived xenograft (PDX) models of squamous cell carcinoma of the uterine cervix. Early and late generation tumors of the BK-12, ED-15, HL-16, and LA-19 model were studied [21, 22].

## RESULTS

### Histological appearance

**Figure 1:**
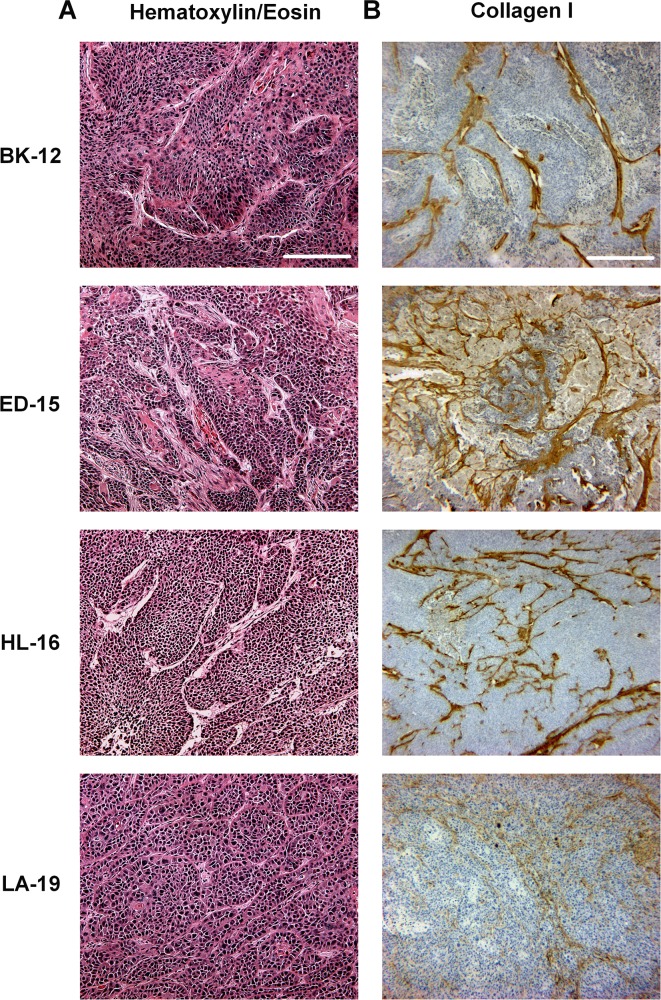
Histological appearance of BK-12, ED-15, HL-16, and LA-19 xenografts **(A)** Tumor preparations stained with hematoxylin and eosin. Scale bar: 200 μm. **(B)** Tumor preparations immunostained for collagen I. Scale bar: 500 μm.

The four PDX models differed considerably in histological appearance, as illustrated by representative images in Figure 1. Tumors of the BK-12, ED-15, and LA-19 model were moderately differentiated, whereas tumors of the HL-16 model were poorly differentiated. The appearance and distribution of connective tissue elements were easily spotted in HE stained sections (Figure 1A) as well as in sections immunostained for collagen I (Figure 1B), with the latter being more suitable for quantitative analyses. Large intra- and intertumor heterogeneity were observed in all four models in terms of collagen I-positive tissue. However, while thick filament bundles were commonly seen in BK-12 and ED-15 xenografts, the collagen fibers in the two other xenograft models – and in LA-19 tumors in particular – were thinner, more numerous, and more dispersed. Early and late generation tumors of the same PDX model were qualitatively similar regarding histological features and collagen I staining.

### DW-MRI results

**Figure 2:**
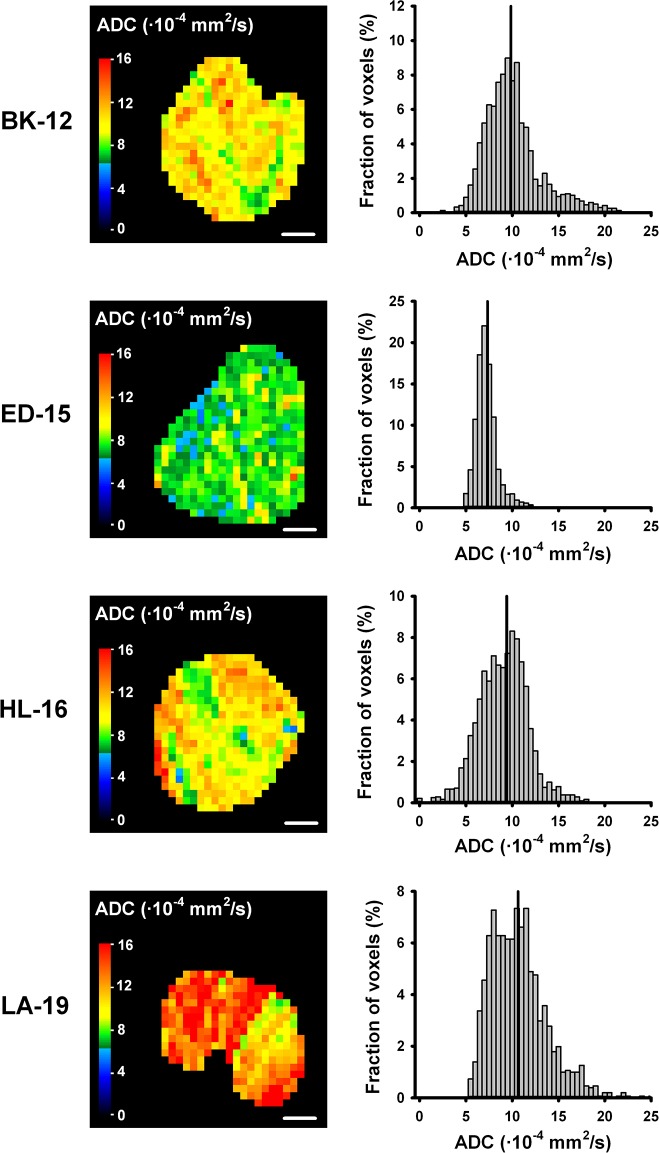
ADC maps and parameter distributions ADC images and frequency distributions of a representative BK-12, ED-15, HL-16, and LA-19 tumor. ADC maps display a central axial tumor section, while frequency distributions include voxel values from the whole tumor. Tumor median values are indicated by vertical lines. Color bars: ADC scales. Scale bars: 2 mm.

DW-MRI was performed with four different diffusion encoding constants (*b*) of 200, 400, 700, and 1000 s/mm^2^ and diffusion gradient sensitization in three orthogonal directions. ADC values were calculated by using a mono-exponential diffusion model. Good fits to experimental data were obtained, with correlation coefficients > 0.90 for all tumors. A non-directional ADC map and parameter distribution for a representative tumor of each PDX model are displayed in Figure 2. Heterogeneity was substantial, both among tumors of the same PDX model and within individual tumors, with high ADC values located in peripheral as well as central tumor regions. Moreover, the shape of the parameter distributions varied notably from tumor to tumor. Qualitatively, no differences could be observed between the ADC maps of early and late generation xenografts of the same tumor model.

### ADC values *versus* collagen I fraction

**Figure 3:**
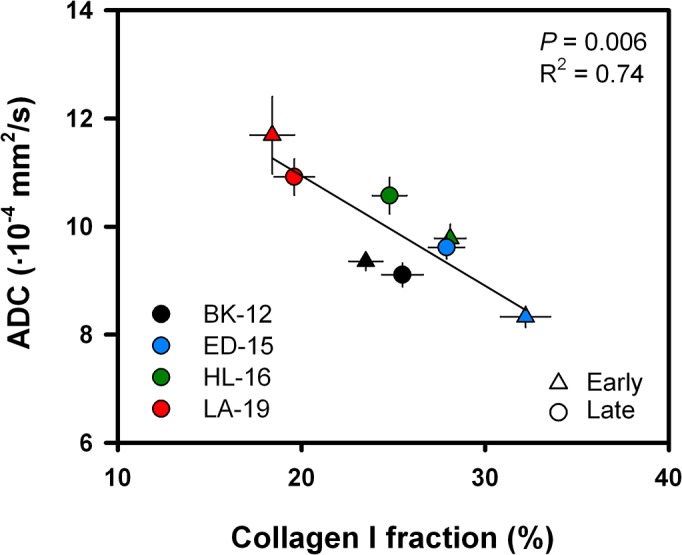
ADC *versus* collagen I fraction Median ADC plotted as a function of collagen I fraction for early generation (triangles) and late generation (dots) tumors of each PDX model. Symbols: mean values ± SEM of 10–28 tumors. Curve: linear regression line.

Figure 3 presents the quantitative relationship between ADC and the fraction of collagen I-positive tumor tissue. Whole tumor median ADC is plotted as a function of collagen I fraction for early and late generation tumors of the PDX models. Data point values are provided in Table 1. A significant inverse correlation was found (*P* = 0.006), a correlation that was well described by a linear regression curve. Non-directional ADC values are presented, as the filament bundles of collagen fibers are expected to be randomly oriented in isotropic tumors like the cervical cancer xenografts of interest. Nevertheless, a statistically significant inverse relationship was also detected between ADC and collagen I fraction for each of the three orthogonal diffusion gradient directions (Supplementary Figure 1). Since different cohorts of tumor-bearing mice were subjected to MRI and histological examinations in these experiments, the association between ADC and collagen I fraction could not be analyzed on an individual tumor level in the current study.

**Table 1:** Collagen I fractions and ADC values

		Collagen I fraction (%)^*^	ADC (·10^−4^ mm^2^/s)^**^
BK-12	Early Late	23.5 ± 0.9 25.5 ± 1.1	9.36 ± 0.17 9.11 ± 0.22
ED-15	Early Late	32.2 ± 1.4 27.9 ± 1.0	8.33 ± 0.20 9.62 ± 0.21
HL-16	Early Late	28.1 ± 0.9 24.8 ± 0.9	9.78 ± 0.26 10.57 ± 0.34
LA-19	Early Late	18.4 ± 1.2 19.6 ± 1.1	11.69 ± 0.72 10.92 ± 0.33

^*^Mean ± SEM of 22–28 tumor values. ^**^Mean ± SEM of 10–15 tumor median values.

## DISCUSSION

Over the last decades, DW-MRI has developed into a powerful method in clinical practice. Even so, the factors and mechanisms governing the diffusion of water in biological tissues remain to be fully understood [2]. The present study sought to elucidate whether DW-MRI-derived ADC values are related to collagen I amount across four PDX models of uterine cervical cancer. These PDX models have been found to be relevant models for human disease in previous experiments in our group, as they reflect essential biological properties of the donor patients’ tumors [21, 22]. Importantly, type I collagen is the most prominent component of the stroma in these xenografts, and the volume of the stromal compartment varies appreciably between the different models [21]. As such, our panel of PDX models is well suited for addressing the hypothesis of current interest. Since fundamental characteristics of PDX models may gradually be altered by a large number of murine serial transplantations, early as well as late generation xenografts were studied.

In imaging-based research, preclinical studies have the advantage over clinical studies that the imaging conditions can be controlled more easily. Besides, multiple tumors of the same genetic basis – representing several copies of an individual patient lesion – can be examined in the same experiment. As preclinical models, PDX models are thought to better recapitulate the biological diversity and therapeutic responsiveness of human cancers than tumor models derived from previously established cell lines [23, 24]. Nevertheless, also PDX models have inherent limitations worthy of attention. Of particular importance in the current context is the fact that only the parenchyma of patient-derived xenografts can be expected to be of human origin after a few serial transplantations in mice [24]. Consequently, the stromal compartment of patient-derived xenografts – as in other tumor xenografts – may not necessarily be representative for that of human tumors. Furthermore, the architecture and composition of the extracellular matrix are results of a dynamic interplay between parenchymal cells, activated connective tissue cells, and different types of immune cells [25]. Because patient-derived xenografts have to be grown in immune-deficient animals like the BALB/c *nu*/*nu* mice, inflammatory responses and, hence, the stromal conditions may differ from the ones seen in spontaneous animal lesions or tumors in man. This could also be the case in the four PDX models of current interest; nonetheless, these models reflect the appearance of the stroma and the inflammatory reaction of their respective donor patient tumor [21, 22].

Tissue diffusivity measurements are intrinsically sensitive to any biophysical process causing movement of molecules. Fluid flow, perfusion, and active transport may all potentially increase the apparent water mobility. Moreover, macroscopic tissue movements (*e.g.* cardiac and respiratory motions) can cause severe image artifacts, making accurate calculation of ADC values problematic. While motion correction algorithms, cardiac and respiratory gating, and signal averaging may help resolve the challenges related to bulk movements, an appropriate choice of *b* values is of crucial importance to minimize microenvironmental effects. In the present study, diffusion sensitization was achieved with four different diffusion encoding constants of 200, 400, 700, and 1000 s/mm^2^. This is in accordance with the recommendations of Padhani *et al.* [1], stating that two or more *b* values should be used for ADC quantification in DW-MRI. Further, the chosen *b* values should be sufficiently high to avoid influence of fluid flow and perfusion (*b* ≥ 100 s/mm^2^) and sufficiently low to diminish effects of intracellular diffusion of water molecules (*b* ≤ 1000 s/mm^2^) [1, 26]. Hence, image contrast can most likely be attributed to extravascular extracellular diffusion phenomena in the current experimental setting.

Over a broad range of *b* values, the DW-MRI signal from biological tissues is known to decay in a multi-exponential manner [27]. However, the data acquired in most clinical applications with *b* values between 100 and 1000 s/mm^2^ are adequately modeled using a mono-exponential diffusion model [1]. Because mono-exponential decay curves yielded good fits to the diffusion data obtained from the PDX models in question, the choice was made not to complexify the analysis method further.

As mentioned introductorily, it is widely accepted that ADC values are sensitive to tumor cellularity [1, 5, 7–10, 27]. Investigations on the possible dependence of ADC values on connective tissue parameters, on the other hand, are less in number. Ko *et al.* [28] found significantly lower ADC values in estrogen receptor-positive breast carcinomas with a collagen-dominant stroma type as compared to carcinomas with a fibroblast- or lymphocyte-dominant stroma type. Also, collagen-rich non-differentiated pancreatic ductal adenocarcinoma (PDAC) xenografts can be distinguished from collagen-poor differentiated PDAC xenografts on the basis of ADC values [29]. Moreover, the study by Hompland *et al.* [20] revealed a significant inverse correlation between ADC and the fraction of collagen-containing connective tissue for xenografts of uterine cervical carcinoma, warranting the experiments described herein. These previous reports, and the current findings on the association between ADC and collagen I fraction across several different PDX models of cervical cancer, add further insight into the complexity of water diffusion in tumor tissue, and suggest that ADC values are not determined solely by the cell density. Thus, there is increasing evidence that collagen fibers in the extracellular space have the ability to inhibit the movement of water molecules, and that this is detectable by DW-MRI.

Qualitative examinations of HE-stained histological sections have uncovered that our PDX models do not differ significantly in tumor cell density [21, 22]. Furthermore, the cell density was equal in early and late generation tumors of the same model. As seen in Table 1, early generation tumors showed higher ADC values and lower collagen I fractions than late generation tumors in the BK-12 and LA-19 models, whereas in the ED-15 and HL-16 models, the ADC values were lower and the collagen I fractions were higher in early than in late generation tumors. Taken together, these observations reduce the probability that other histomorphological parameters than stromal content have confounded our data, and contribute to explain why ADC values do not reflect cellularity in all tumors [1, 13].

Clinical investigations have provided some evidence that low pretreatment ADC values are associated with tumor aggressiveness and poor treatment outcome in cervical carcinoma [30–32]. BK-12 and LA-19 tumors are highly aggressive and develop lymph node metastases frequently while ED-15 and HL-16 tumors do not metastasize readily [21, 22], and consequently, there is no association between ADC and metastatic propensity in these cervical cancer models. This observation is not unexpected because it is well established that lymph node metastasis in cervical carcinoma is associated with other microenvironmental parameters than high cellularity and a dense extracellular matrix, including extensive tumor hypoxia, elevated interstitial fluid pressure, and high lactate concentrations [33–38]. Furthermore, the high metastatic propensity of BK-12 and LA-19 tumors has been revealed to be associated with the presence of functional intratumoral lymphatics [22].

The high cellularity and dysfunctional vasculature commonly found in malignant lesions give rise to tumor hypoxia. In the search for robust biomarkers of hypoxia-related parameters in cervical cancer, dynamic contrast-enhanced (DCE) MRI has shown promise [39–42]. Former experiments with the BK-12, ED-15, HL-16, and LA-19 models in our laboratory have evinced strong correlations between DCE-MRI-derived parameters and the fraction of hypoxic tumor tissue [43]. DW-MRI and DCE-MRI are applicable in the same MR examination, and it is thus possible that these non-invasive imaging methods may complement each other in the characterization of the tumor microenvironment.

The clinical value of DW-MRI has been demonstrated by a large number of studies, including investigations on uterine cervical cancer. Evidence has been provided that DW-MRI can help differentiate malignant and benign cervical tissues and detect pelvic lymph node metastases [44–46]. Besides, ADC has proven to be a promising biomarker for treatment-induced tumor cell death and disease outcome [32, 47]. The observations reported in the present communication may contribute to further enhance the clinical utility of this non-invasive imaging technique. For instance, as tumors with a particularly stiff extracellular matrix are likely to exhibit a more aggressive phenotype [25], DW-MRI could be useful in treatment stratification, by identifying those patients in need of additional therapy. Further, the sensitivity of DW-MRI to potential delivery barriers to therapeutic agents in the tumor stroma provides added value to the use of ADC as a predictive biomarker for response to cancer therapy. Also, DW-MRI could play an important role in the development of drugs aiming to modify the stromal microenvironment in tumors, such as inhibitors of signaling molecules and remodeling enzymes like the sonic hedgehog (Shh), focal adhesion kinase (Fak), and lysyl oxidase-like-2 (LOXL2) [48, 49].

To summarize, the current preclinical study demonstrates that essential information on the connective tissue fraction in tumors can be obtained by DW-MRI. Across four biologically dissimilar PDX models of cervical cancer, representing four different patient tumors, we found a significant inverse correlation between ADC and the fraction of collagen I-positive tissue. Alongside the increased acknowledgement of the importance of the tumor stroma in cancer development and for the outcome of treatment, these results may prove valuable in a clinical setting.

## MATERIALS AND METHODS

### PDX models

Four PDX models (BK-12, ED-15, HL-16, and LA-19) of squamous cell carcinoma of the uterine cervix, derived from patients with FIGO stage IIB disease, were included in the study [21, 22]. Adult (8–12 weeks of age) female BALB/c *nu*/*nu* mice were used as host animals. Two frozen cell stocks of these PDX models have been established in our group, one from xenografted tumors in passage 2 (early generation) and the other from xenografted tumors transplanted serially in mice for approximately two years (late generation). The experiments described herein were performed with early generation as well as late generation xenografts. Aliquots of 5 × 10^5^ cells, obtained from intramuscular tumors initiated from the frozen stocks, were inoculated in the left *quadriceps femoris* of mice for tumor initiation. Xenografts of volume 150–1500 mm^3^ were included in the experiments. Animal care and experimental procedures were approved by the Institutional Committee on Research Animal Care and were performed in agreement with the Interdisciplinary Guidelines for the Use of Animals in Research, Marketing, and Education (New York Academy of Sciences, New York, NY, USA).

### Histological examination

Histological sections were prepared by standard procedures and stained with hematoxylin and eosin (HE) or immunostained for collagen I. An anti-collagen I rabbit polyclonal antibody (Abcam, Cambridge, UK) was used as primary antibody. For each of 22–28 early or late generation tumors per PDX model, quantitative studies were carried out on three sections cut through central tumor regions. The fraction of collagen I-positive tissue, defined as the area fraction of non-necrotic tissue showing positive staining for collagen I, was assessed by image analysis.

### Magnetic resonance imaging

Mice were imaged with a Bruker Biospec 7.05 T bore magnet (Bruker Biospin, Ettlingen, Germany), situated at the MRI Core Facility for Preclinical Cancer Research, Oslo University Hospital. A mouse quadrature volume coil was utilized. For each PDX model, 10–15 early or late generation tumors were scanned with axial slices covering the entire tumor volume. Mice were anesthetized with ∼4.0 % Sevofluran in O_2_ (Baxter, IL, USA) at a flow rate of 0.5 l/min during scanning. Respiration rate and body core temperature were monitored continuously with a pressure sensitive abdominal probe and a rectal temperature probe (Small Animal Instruments, New York, NY, USA), respectively. The gas anesthesia was adjusted manually to maintain a stable respiration rate, whereas automated hot air flow regulation kept the body core temperature at 37°C.

A fast spin echo pulse sequence (RARE) with a repetition time (TR) of 2500 ms, an echo time (TE) of 35 ms, an image matrix of 128 × 128, a field of view (FOV) of 3 × 3 cm^2^, a slice thickness of 0.7 mm, a slice gap of 0.3 mm, two averages, and fat suppression was applied to acquire anatomical *T*_2_-weighted images. DW-MRI was accomplished as previously described [50]. In brief, a diffusion-weighted single-shot fast spin echo pulse sequence (RARE) with a TR of 1300 ms, a TE of 26 ms, an image matrix of 64 × 64, a FOV of 3 × 3 cm^2^, a slice thickness of 0.7 mm, a slice gap of 0.3 mm, and fat suppression was used. Diffusion sensitization was achieved with four different *b* values of 200, 400, 700, and 1000 s/mm^2^, a diffusion gradient duration of 7 ms, and a diffusion gradient separation time of 14 ms, in three orthogonal directions. Analysis of DW images was performed on a voxel-by-voxel basis using tumor regions of interest (ROIs) delineated in the *T*_2_-weighted images. In-house-made software developed in Matlab (MathWorks, Natick, MA, USA) was utilized to calculate directional and non-directional ADC values for each voxel, and thereby produce parametric ADC maps. The following mono-exponential model equation for signal intensity (*S*) was employed:

ln(S(b)) = –b⋅ADC + c

where *c* equals the natural logarithm of the signal intensity without diffusion sensitization, *i.e.* ln (*S*(*b* = 0)) [1]. In the subsequent analysis, the median non-directional ADC value of the whole tumor was considered.

### Statistical analysis

Statistical analysis was carried out with the SigmaStat statistical software package (Systat Software Inc., San Jose, CA, USA). Regression analysis and the Pearson product moment correlation test were used to study the relationship between ADC values and the fraction of collagen I-positive tumor tissue. Probability values of *P* < 0.05 were regarded as statistically significant.

## SUPPLEMENTARY FIGURE


