# Supplementary material for: Diffusion-weighted MRI-derived ADC values reflect collagen I content in PDX models of uterine cervical cancer
Source: Oncotarget. 2017 Nov 11;8(62):105682–91. doi: 10.18632/oncotarget.22388 (PMC5739670; doi:10.18632/oncotarget.22388)
Supplement: Supplementary file 1 [file oncotarget-08-105682-s001.pdf]

Diffusion-weighted MRI-derived ADC values reflect collagen I content in PDX models of uterine cervical cancer

SUPPLEMENTARY MATERIALS

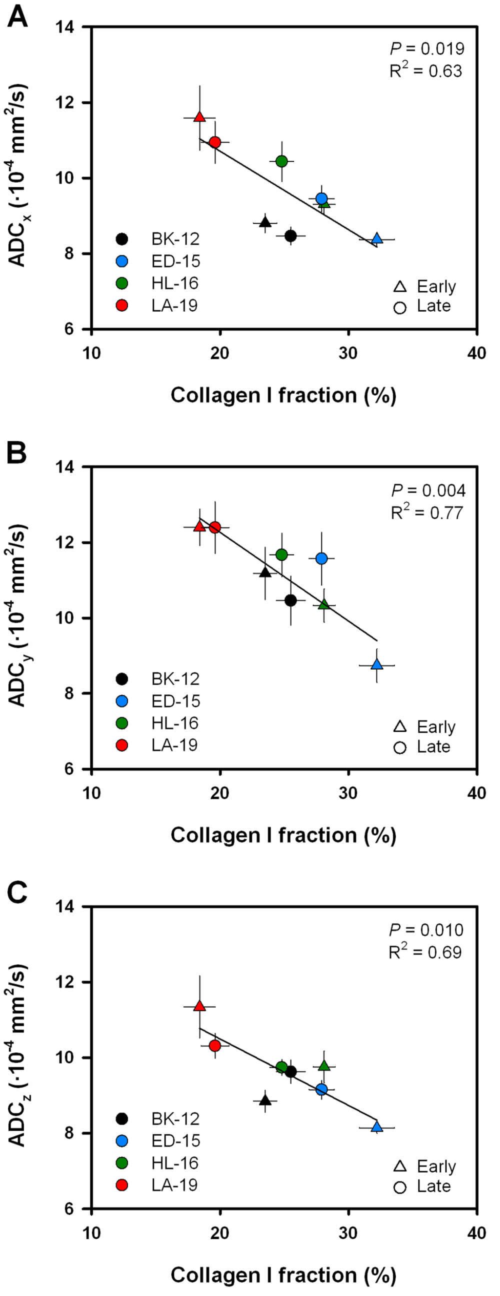

Supplementary Figure 1: ADC *versus* collagen I fraction for each diffusion gradient direction. ADC_x **(A)**, ADC_y **(B)**, and ADC_z **(C)** plotted as a function of collagen I fraction for early generation (triangles) and late generation (dots) tumors of each PDX model. Symbols: mean ± SEM of 10–28 tumor median values. Curves: linear regression lines.
